# Validation of Cell-Free RNA and Circulating Tumor Cells for Molecular Marker Analysis in Metastatic Prostate Cancer

**DOI:** 10.3390/biomedicines9081004

**Published:** 2021-08-12

**Authors:** Michael Ladurner, Manuel Wieser, Andrea Eigentler, Martin Seewald, Gabriele Dobler, Hannes Neuwirt, Mona Kafka, Isabel Heidegger, Wolfgang Horninger, Jasmin Bektic, Helmut Klocker, Peter Obrist, Iris E. Eder

**Affiliations:** 1 Department of Urology, Medical University Innsbruck, 6020 Innsbruck, Austria; michael.ladurner@tirol-kliniken.at (M.L.); andrea.eigentler@i-med.ac.at (A.E.); gabriele.dobler@tirol-kliniken.at (G.D.); mona.kafka@i-med.ac.at (M.K.); isabel-maria.heidegger@i-med.ac.at (I.H.); wolfgang.horninger@i-med.ac.at (W.H.); jasmin.bektic@tirol-kliniken.at (J.B.); helmut.klocker@i-med.ac.at (H.K.); 2 Tyrolpath Obrist Brunhuber GmbH, 6511 Zams, Austria; manuel.wieser@tyrolpath.at (M.W.); martin.seewald@tyrolpath.at (M.S.); peter.obrist@tyrolpath.at (P.O.); 3 Department of Internal Medicine IV-Nephrology and Hypertension, Medical University of Innsbruck, 6020 Innsbruck, Austria; hannes.neuwirt@i-med.ac.at

**Keywords:** metastatic prostate cancer, circulating tumor cells, cfRNA, castration resistant prostate cancer

## Abstract

Since tissue material is often lacking in metastatic prostate cancer (mPCa), there is increasing interest in using liquid biopsies for treatment decision and monitoring therapy responses. The purpose of this study was to validate the usefulness of circulating tumor cells (CTCs) and plasma-derived cell-free (cf) RNA as starting material for gene expression analysis through qPCR. CTCs were identified upon prostate-specific membrane antigen and/or cytokeratin positivity after enrichment with ScreenCell (Westford, Massachusetts, USA) filters or the microfluidic Parsortix^TM^ (Guildford, Surrey, United Kingdom) system. Overall, 50% (28/56) of the patients had ≥5 CTCs/7.5 mL of blood. However, CTC count did not correlate with Gleason score, serum PSA, or gene expression. Notably, we observed high expression of *CD45* in CTC samples after enrichment, which could be successfully eliminated through picking of single cells. Gene expression in picked CTCs was, however, rather low. In cfRNA from plasma, on the other hand, gene expression levels were higher compared to those found in CTCs. Moreover, we found that *PSA* was significantly increased in plasma-derived cfRNA of mPCa patients compared to healthy controls. High PSA expression was also associated with poor overall survival, indicating that using cfRNA from plasma could be used as a valuable tool for molecular expression analysis.

## 1. Introduction

Prostate cancer (PCa) is the most frequent male cancer in Western societies followed by lung and colon cancer [1]. Disease stages range from slow-growing local tumors to aggressive stages with high metastatic potential [2]. Because of the strong relevance of androgens, androgen deprivation therapy (ADT) is a mainstay in the treatment of metastatic prostate cancer (mPCa). Despite high response rates, however, progression to castration-resistant prostate cancer (CRPC) eventually occurs in nearly all patients [3]. Still, mPCa is not curable; however, the number of available therapies to slow tumor progression significantly increased in the past few years, including chemotherapy even in earlier hormone-sensitive settings, anti-androgenic drug abiraterone [4], androgen receptor inhibitors like enzalutamide, apalutamide and darolutamide [5,6,7], and radium-223 [8]. Most recently, poly (adenosine diphosphate-ribose) polymerase (PARP) inhibitors have been approved for the treatment of metastatic CRPC (mCRPC) but were limited to use in patients with verified mutations of DNA repair genes (*BRCA1*, *BRCA2*) who have progressed following prior treatment with a new hormonal agent [9]. This increasing selection of drugs is certainly an overall improvement in the treatment of mPCa; however, most of these therapies are only effective in a limited proportion of patients. PARP inhibitors, for example, can only be used in an estimated number of 10–15% of men. Moreover, a substantial number of patients do not respond to second-generation antiandrogens or develop resistances over time (reviewed by [10]). Thus, treatment of mPCa will develop into a more sophisticated individualized therapy in the future, which requires valid markers to help decision making. Still, the primarily used biomarker to monitor therapy response is serum prostate-specific antigen (PSA), which, however, only hardly reflects tumor burden in CRPC [11]. 

There is accumulating interest in the use of liquid biopsies such as circulating tumor cells (CTCs) and circulating cell-free (cf) nucleic acids (DNA or RNA), which can be easily taken at all stages of mPCa with minimal intervention. An increased number of CTCs (≥5 cells/7.5 mL) in the blood of patients with mPCa has been previously associated with tumor progression [12]. Furthermore, CTC count was shown to be useful to monitor therapy responses [13,14,15]. Besides determining CTC count, CTCs have also been used to screen for genetic and molecular aberrations such as androgen receptor (*AR*) amplifications [16], AR overexpression, status of the ETS-related gene (*ERG*) and the phosphatase and tensin homolog (*PTEN*) [17], and the occurrence of the *AR* variant *AR-V7* that predicts limited response to enzalutamide and abiraterone [18,19,20]. These data highlight the usefulness of CTCs to analyze possible molecular changes in tumor cells of mPCa where representative tissue material is often lacking. Despite these promising data, however, liquid biopsies—in particular CTCs—have not yet reached a universally accepted use in the clinical management of PCa patients. One possible reason may be the challenge to efficiently isolate CTCs from the blood which requires a two-step technology based on enumeration and specific detection of CTCs among contaminating blood cells, which is often hampered due to the heterogeneous expression of the markers used for CTC recognition. For the isolation of CTCs in PCa, currently only the CellSearch system (Menarini, Silicon Biosystems, Bologna, Italy) has been approved by the FDA. This technology identifies CTCs based on the expression of EpCAM, which is often lost during tumor progression (reviewed by [21]). To prevent the restricted analysis of a specific EpCAM-positive CTC population, which might weaken the results, other marker-independent systems have been established (reviewed by [22,23]). Among them are various filtration systems such as ScreenCell, where the enrichment of CTCs is mainly based on isolation by size, or microfluidic methods such as Parsortix™ (ANGLE plc, Guildford, Surrey, UK), where cells are additionally selected upon deformability and which has recently been tested in PCa [24].

Another possibility to screen for molecular alterations in metastatic disease is the use of blood-derived circulating cfDNA or cfRNA. Several studies reported on the detectability of *AR-V7* in whole blood mRNA or RNA from exosomes [19,25,26,27]. Moreover, Kohli et al. determined *AR* gene amplifications and mutations in DNA repair genes in circulating tumor (ct) DNA of mPCa [28], and Fettke et al. demonstrated the simultaneous detection of *AR* alterations in cfDNA and cfRNA useful to guide treatment in advanced PCa [29]. The isolation of blood-derived cf nucleic acids is relatively cheap and easy compared to CTCs, though it is not trivial to discriminate between nucleic acids shed from normal or tumor cells. Taken together, CTCs as well as cf nucleic acids have their strengths and limitations in their use as biomarkers. Both have been shown to be valuable tools in mPCa, but the question remains if one or the other technique should be favored. The aim of this study was to determine CTC count and the expression of a pre-selected gene panel either in CTCs or plasma-derived cfRNA from patients with high metastatic tumor load at different tumor stages and various prior therapies.

## 2. Materials and Methods

### 2.1. Patients’ Characteristics

Between March 2017 and December 2019, we collected 62 blood samples from patients with histologically and radiographically confirmed mPCa. Patients were between 56 and 88 years old and had a median serum PSA of 46.3 ng/mL. The clinical characteristics of the patients are summarized in Table 1. Patients were recruited in our outpatient clinic with different prior therapies ranging from newly diagnosed mHSPC with ADT to mCRPC patients undergoing 4th line therapy. One patient was monitored over 6 months with 5 consecutive blood draws. According to the Advanced Prostate Cancer Consensus Conference (APCCC) of 2015, tumor progression (TU progress) in mCRPC with serum testosterone <50 ng/dL was defined as at least two out of the following three criteria: PSA rise, radiographic progression, or clinical progression [30]. Blood samples from healthy donors (n = 3) were collected at the Department of Urology, Medical University Innsbruck, Austria, and served as healthy controls. The study was carried out in accordance with ethical approval from the Medical University Innsbruck (Approval Number 2014-0021, UN4837). Written informed consent was obtained from all participants prior to blood draw.

### 2.2. Enumeration and Counting of Patient Derived CTCs

CTCs were isolated either with the size-based ScreenCell filtration system or the deformability-based microfluidic Parsortix™ (ANGLE plc, Guildford, UK) technology. Blood from the first 34 patients was collected into Streck tubes (Cell-free DNA BCT^®^CE Streck, La Vista, NE, USA), and 3 mL of blood was diluted with 4 mL FC2 buffer (ScreenCell, Sarcelles, France) and subsequently filtered through ScreenCell Cyto within 2 h. Then, filters were dried at room temperature. From a second cohort of patients (n = 28), 5 mL of blood that was collected into TransFix tubes (CTC-TVTs, CYTOMARK, Buckingham, UK) was pumped through the Parsortix™ cassette. The restrained cells were stained with a mixture of an Alexa Fluor^®^ 488 anti-human prostate-specific membrane antigen (PSMA) antibody (4 µg/mL) (BioLegend, #342506, clone LNI-17), an Alexa Fluor^®^ 488 anti-human pan-cytokeratin (panCK) antibody (5 µg/mL) (BioLegend, San Diego, CA, USA), and an Alexa Fluor^®^ 647 anti-human CD45 antibody (12.5 µg/mL) (Sony Biotechnology, San Jose, CA, USA). Nuclei were stained with DAPI to indicate the presence of a nucleus. Putative CTCs were counted when they were positive for PSMA and/or panCK and negative for CD45 using a Zeiss Axio Observer A1 (Zeiss Microscope, Jena, Germany). Final CTC numbers were extrapolated to 7.5 mL of blood.

### 2.3. Picking Single CTCs

To pick single CTCs, cells were enriched with the Parsortix^TM^ device and then transferred into a microwell plate (Sievewell, Sanukawa, Kanagawa, Japan, 20 µm width, 25 µm depth) where they were stained for PSMA, panCK, CD45, and DAPI. PSMA and/or panCK positive CTCs and CD45^+^ white blood cells (WBCs) were picked in a volume of 7 µL PBS using the ALS CellCelector™ automated rare single cell picking system (Jena, Germany), directly transferred into 7 µL RNA lysis buffer, and immediately stored at −80 °C.

### 2.4. RNA Isolation from CTCs and Plasma

To isolate cfRNA from plasma, blood was withdrawn into standard 9 mL ethylenediaminetetraacetic acid (EDTA) vacutainers (Beckton Dickinson, Heidelberg, Germany) and processed within 2 h. In brief, whole blood was centrifuged at 1900 *g* for 10 min at 4 °C. The supernatant was centrifuged again at 16,000 *g* for 10 min at 4 °C to remove cell debris and plasma aliquots were stored in 2 mL DNA LoBind tubes (Eppendorf, Hamburg, Germany, PCR clean, safe-lock) at −80 °C until RNA extraction. RNA was isolated from 2 mL of plasma with the RNeasy^®^ Plus Micro Kit (Qiagen, Hilden, Germany) according to the manufacturer’s instructions with a final elution volume of 30 µL. To isolate RNA from CTCs, we used the RNeasy Plus Micro Kit (Qiagen) according to the manufacturer’s instructions with a final elution volume of 14 µL. To isolate RNA from CTCs enriched with ScreenCell, 6 mL of blood was diluted with 1 mL LC dilution buffer (ScreenCell) as suggested by the manufacturer and filtered through ScreenCell Cyto (ScreenCell) within 2 h after blood draw. Capsule filters were then inserted into 1.5 mL DNA LoBind tubes (Eppendorf) and 100 µL RNA lysis buffer (RNeasy Plus Micro Kit, Qiagen) containing 4 ng/µL carrier RNA (RNeasy Plus Micro Kit, Qiagen) was added. Tubes were centrifuged at 12,000 *g* for 1 min, and the flow-through was stored at −80 °C until further processing. To isolate RNA from unpicked CTCs, blood was processed through the Parsortix^TM^ device, and the chip was floated with RNA lysis buffer containing 4 ng/µL carrier RNA (RNeasy Plus Micro Kit, Qiagen, Germantown, MD, USA).

### 2.5. cDNA Synthesis, Pre-Amplification, and Real-Time Quantitative RT-PCR (qPCR)

Before transcription into cDNA using the iScript gDNA Clear cDNA Synthesis Kit (Biorad, Hercules, CA, USA), RNA was digested with DNase to remove genomic DNA contamination. Then, cDNA was pre-amplified with SSoAdvanced PreAmp Supermix (Biorad) using a pool of primers as listed in Table 2. qPCR was carried out with ABI Prism 7500 Fast RT-PCR System (Applied Biosystems, Waltham, MA, USA) cycler as previously described [31] and run over 40 PCR cycles. Gene expression was confirmed positive when both duplicates of each sample revealed detectable Ct (cycle threshold) values (≤39). For method establishment and validation of gene expression, we conducted the same protocol using 2.5 ng RNA from immortalized cell lines for pre-amplified cDNA. Primer sequences for qPCR were listed in Table 2. Ct levels from genes of interest (GOI) were normalized with *TBP* using the mathematical model ratio 2^−ΔCt^ (dCt = Ct gene of interest−Ct *TBP*).

### 2.6. Cell Culture

Control experiments were performed with three immortalized prostate cancer cell lines (VCaP, PC-3, and LAPC4enza). VCaP and PC-3 cells were obtained from the American Type Culture Collection (ATCC, Rockville, MD, USA) and cultured in RPMI 1640 (Lonza, Basel, Switzerland) supplemented with 10% fetal calf serum (FCS) (Gibco, Grand Island, NY, USA), 1% penicillin/streptomycin (Lonza), and 1× GlutaMAX^TM^ (Gibco). LAPC4enza was recently established in our lab through long-term culture of LAPC4 in RPMI 1640 supplemented with 10% FCS (Gibco), 1% penicillin/streptomycin (Lonza), 1× GlutaMAX^TM^ (Gibco), and 8 µM enzalutamide [32]. These three cell lines were chosen because of their expression of AR and PSMA: VCaP (AR-FL^+^/AR-V7^+^, PSMA^+^), PC-3 (AR-FL^−^/AR-V7^+^, PSMA LAPC4enza (AR-FL^+^/AR-V7^+^, PSMA^+^). Cells were grown in 75 cm^2^ flasks until about 80% confluency.

### 2.7. Statistical Analysis

Statistical differences were calculated using SPSS (V15.0 and V26). Proportions were compared using the Chi2 or Fisher exact tests. Non-parametric tests (Mann–Whitney U) were used to compare continuous variables. Factors potentially associated with overall survival were assessed using Kaplan–Meier plot and Log-Rank test. Compared groups are given in the figures and/or figure legends, and significances are encoded as follows: * *p* < 0.05; ** *p* < 0.01; *** *p* < 0.001. Data are presented as mean plus standard error of the mean (SEM) from three independent experiments unless otherwise stated. 

## 3. Results

### 3.1. CTC Count Did Not Correlate with Gleason Score or Serum PSA Levels

**Figure 1:**
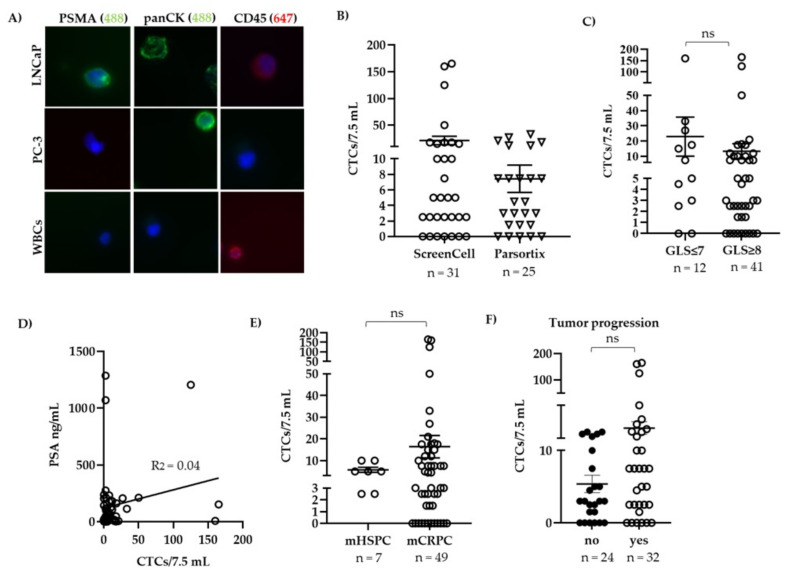
CTC count in patients with metastatic prostate cancer. (**A**) LNCaP, PC-3 cells, and white blood cells (WBCs) were stained for prostate-specific membrane antigen (PSMA), pan-cytokeratin (panCK), and CD45 by immunofluorescence. (**B**) Blood from patients was processed through ScreenCell or Parsortix^TM^ for CTC enrichment. CTCs were counted when they were positive for PSMA and/or panCK and negative for CD45 and expressed as CTCs/7.5 mL of blood. CTC count was then correlated to (**C**) low and intermediate risk (Gleason score, GLS ≤ 7) vs. high risk PCa (GLS ≥ 8), (**D**) serum PSA at the time of blood draw for CTC counting, (**E**) metastatic hormone-sensitive prostate cancer (mHSPC) vs. metastatic castration-resistant prostate cancer (mCRPC), and (**F**) tumor progression. ns = not significant (*p* > 0.05).

In this study, we determined the CTC count in 56 blood samples from 41 patients with mPCa diagnosed through imaging. As summarized in Table 1, our study cohort was very heterogeneous comprising different tumor stages (hormone-sensitive and castration-resistant mPCa) and various prior therapies. Patients had a median age of 71.1 years (range: 56–88 years) and a median serum PSA of 46.3 ng/mL (range: 0.6–7014 ng/mL). To enrich for CTCs, we used either the size-based ScreenCell^®^ system or the microfluidic Parsortix™ device (ANGLE plc). Six blood samples could not be processed because of technical problems such as heavy blood clotting (n=3), which hampered CTC enumeration, or distortion of filters (n = 3), which impaired valid counting of positive cells. Of note, these technical dropouts were not allied to either of the two enumeration systems. CTCs were identified by immunofluorescent staining when they were positive for PSMA and/or panCK and negative for the leukocyte marker CD45. Because of the heterogeneous expression of PSMA on PCa cells, we simultaneously stained for panCK to enable a broader CTC capture efficiency. As expected, LNCaP cells were positive for PSMA, whereas PC-3 cells were negative (Figure 1A). PanCK, on the other hand, was nicely detected in both cell lines. WBCs were negative for PSMA and panCK but positive for CD45. Unexpectedly, we also observed a faint positive CD45 staining in LNCaP cells.

Overall, we detected at least 1 CTC/7.5 mL in 78.6% (44/56) of the samples (Table 3) with a mean CTC number of 15.1/7.5 mL (range 1–165 CTCs/7.5 mL). In 50.0% (28/56) of the samples, ≥ 5 CTCs/7.5 mL were detected, reaching the clinically prognostic cut-off value that was previously defined for mPCa in the study of DeBono et al. [33]. Mean CTC count was higher in blood of patients which was processed with ScreenCell (21.2 CTCs/7.5 mL) compared to that obtained with Parsortix^TM^ (7.4 CTCs/7.5 mL) (Figure 1B). 

Although a substantial number of samples exhibited high CTC counts (≥5 CTCs/7.5 mL), we did not find a correlation with Gleason score (Figure 1C) or serum PSA (Figure 1D). Notably, mean CTC count was higher in patients with mCRPC (mean CTCs/7.5 mL blood = 16.4 ± 4.9, n = 49) compared to mHSPC patients (mean CTCs/7.5 mL blood = 5.7 ± 1.1, n = 7). In order to analyze more balanced groups with respect to sample number, we next categorized the patients into those with tumor progression and those without as defined under Materials and Methods. As shown in Figure 1F, mean CTC count was higher in patients with tumor progression (22.3 ± 7.7 CTCs/7.5 mL, n = 32) compared to those without (5.8 ± 1.1 CTCs/7.5 mL, n = 24), although this difference was again not statistically significant. There was also no correlation of CTC count to any kind of therapeutic regimen (Supplementary Figure S1), though the number of patients in each group was quite low, hampering reliable statistics. 

### 3.2. Gene Expression in Patient-Derived CTCs

**Figure 2:**
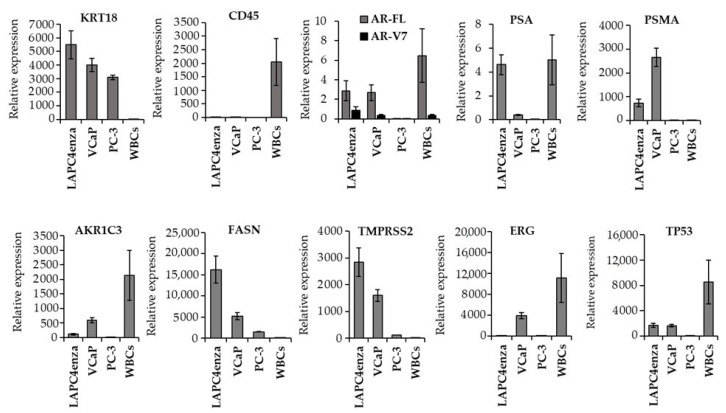
Validation of gene expression. Expression of selected pre-defined genes was analyzed in two AR-FL- and AR-V7-positive cell lines (LAPC4enza, VCaP), in one AR-negative cell line (PC-3), and in white blood cells (WBCs), which were picked from healthy blood upon CD45 positivity and pooled (n = 10). cDNA was amplified prior to qPCR. Ct values were normalized to the housekeeping gene *TBP* (relative expression). Graph shows mean relative expression of GOIs ± SEM.

We next investigated if CTCs could be used for gene expression analysis through quantitative (real time) reverse transcriptase polymerase chain reaction (qRT-PCR). To this end, we pre-defined a specific gene panel, consisting of genes which have been previously associated with PCa: *AR-FL* (androgen receptor-full length), *AR-V7* (variant 7), *PSMA* (prostate-specific membrane antigen), *KLK-2* (kallikrein-2), *PSA* (prostate specific antigen), *AKR1C3* (aldo-keto reductase 1 C3), *ERG* (ETS-related gene), *TMPRSS2* (transmembrane protease, serine 2), *FASN* (fatty acid synthase), and *TP53* (tumor protein 53). In addition, we included two housekeeping genes (TATA-binding protein, *TBP*, and Hypoxanthine phosphoribosyl-transferase 1, *HPRT1*) to enable a normalization of cycle threshold (Ct) values of each gene of interest (GOI) to a constitutively expressed reference gene. Furthermore, we analyzed the expression of two cell type specific genes, the epithelial marker keratin 18 (KRT18) and the leukocyte specific marker gene *CD45 (PTPRC)*, to differentiate between tumor epithelial cells and WBCs. The qPCR protocol was validated with three immortalized PCa cell lines (LAPC4enza, VCaP, PC-3) and a pool of WBCs (n = 10) that were picked from healthy blood after processing through Parsortix^TM^ and staining for CD45. cDNA was amplified prior to qPCR. Ct values of GOIs were normalized with *TBP*. As summarized in Figure 2, the epithelial marker *KRT18* was substantially expressed in all three PCa cell lines but not in WBCs, whereas the leukocyte marker *CD45* was highly expressed in WBCs but absent in the tumor cell lines. *AR-FL*, *AR-V7*, and *PSA* were detected in AR-positive LAPC4enza and VCaP cells and, unexpectedly, also at significant amounts in WBCs. Similarly, *AKR1C3*, *ERG*, and *TP53* were expressed in WBCs. *PSMA*, *FASN*, and *TMPRSS2*, on the other hand, were only detected in the PCa cell lines.

**Figure 3:**
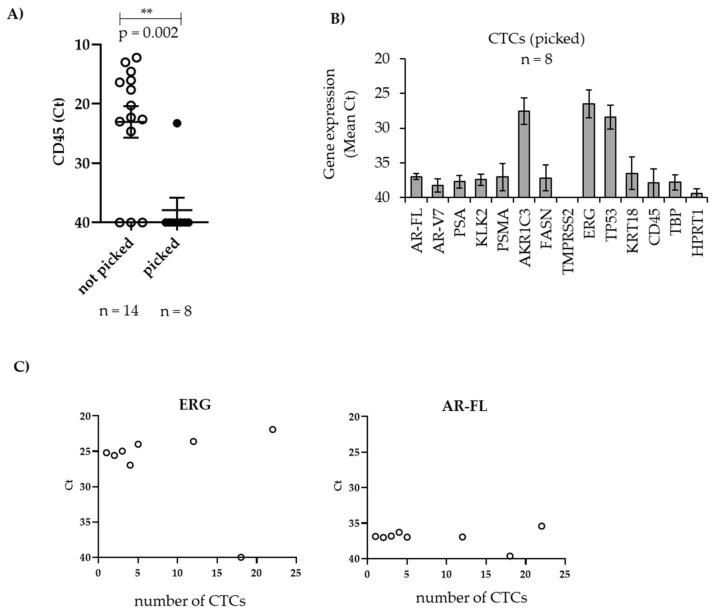
Gene expression in patient-derived CTCs after selective picking of single cells. CTCs were enriched with Parsortix^TM^. (**A**) All restrained cells (not picked) or only PSMA^+^ and/or panCK^+^/CD45^−^ cells, which were selectively picked with the ALS CellCelector, were used for RNA isolation. cDNA was amplified prior to qPCR. Expression (Ct values) of CD45 in unpicked and picked CTCs. (**B**) Gene expression in picked CTCs of different patient samples (n = 8). (**C**) Gene expression using increasing numbers of CTCs. After picking, all CTCs (1–22) from one patient sample were pooled. Statistical comparisons are expressed with asterisks (** *p* < 0.01).

We next looked at the expression of our pre-selected gene panel in CTCs of patients with mPCa. To this end, blood was processed either through ScreenCell (n = 10) or Parsortix^TM^ (n = 4). Following enrichment of CTCs, all restrained cells were transferred to RNA lysis buffer and used to analyze the expression of *CD45* by qPCR to determine contamination of samples with WBCs. As shown in Figure 3A, the majority of samples (11/14) exhibited high expression of CD45 with Ct values ranging from 12.2 to 24.6. To overcome this strong contamination with CD45^+^ cells, we next specifically picked PSMA^+^/panCK^+^/CD45^−^ CTCs using the ALS CellCelector system. Notably, picking of cells was only possible when blood was processed with the Parsortix^TM^ device. We were able to pick 1 to 22 cells per sample (n = 8) with an overall cell picking rate of 52% (Table 4).

Though this cell picking strategy significantly decreased *CD45* expression, all other genes from our pre-defined panel except *AKR1C3*, *ERG*, and *TP53* exhibited low expression with mean Ct values ranging between 35 and 40 (Figure 3B). Even the housekeeping genes *TBP* and *HPRT1* were hardly detectable in the majority of the samples so that a normalization of Ct values to *TBP* was not feasible. Notably, however, Ct values did not decrease with increasing numbers of picked CTCs that were pooled for qPCR. As shown in Figure 3C, strongly expressed *ERG* (mean Ct = 26.5) and weakly expressed *AR-FL* (mean Ct = 30.1) were detectable even in one single CTC, and Ct values were similar irrespective of the number of CTCs used for qPCR, indicating that gene expression levels do not increase when more CTCs were available for qPCR, at least in a range of 1–22 CTCs.

**Figure 4:**
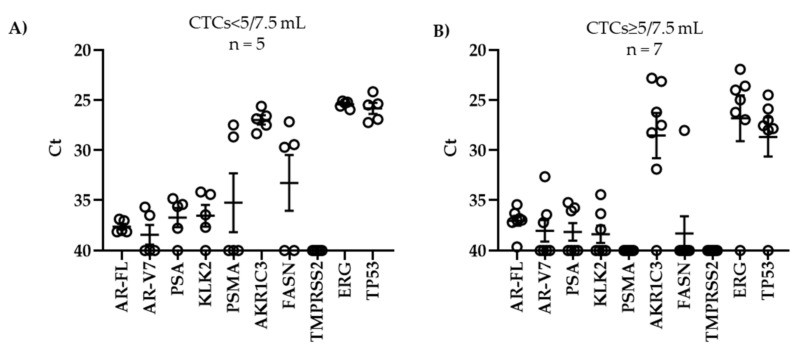
Gene expression in CTCs in correlation with CTC count. After enrichment of CTCs with Parsortix^TM^, RNA was isolated from CTCs, and cDNA was amplified for qPCR. (**A**,**B**) Ct values were depicted in correlation with CTC count. Mean values with SEM are indicated.

When we evaluated gene expression in our patient-derived CTC samples using Ct values, we observed a strong heterogeneity of expression among the genes (Figure 4). *AKR1C3*, *ERG*, and *TP53* exhibited low Ct values, indicating high expression, whereas *AR-FL*, *AR-V7*, *PSA*, and *KLK2* were only weakly expressed. Of note, *AKR1C3*, *ERG*, and *TP53* were also detected at high levels in WBCs (Figure 2). *TMPRSS2* was not detected in any CTC sample. As shown in Figure 4, we did not find any significant differences in gene expression between patients with ≥ 5 CTCs/7.5 mL and those with < 5 CTCs/7.5 mL, though the number of analyzed samples was low.

### 3.3. Gene Expression in Plasma-Derived cfRNA

**Figure 5:**
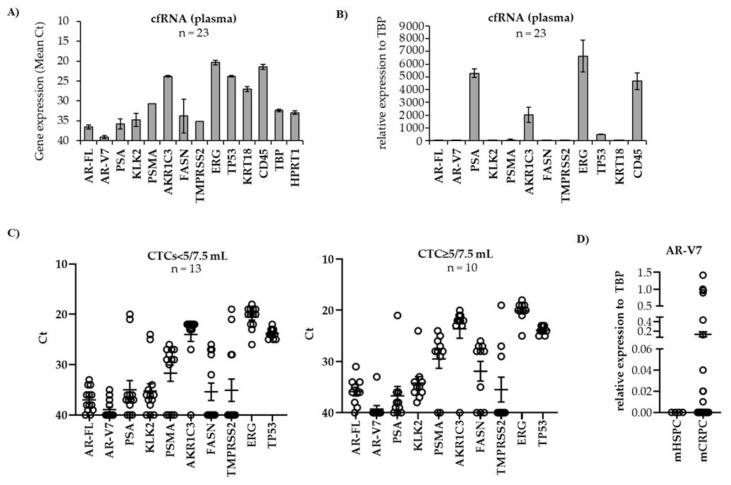
Gene expression in plasma-derived cfRNA. cfRNA was isolated from plasma of mPCa patients as described in Material and Methods. cDNA was amplified prior to qPCR. Gene expression was depicted as (**A**,**C**) mean Ct values for each individual gene or (**B**) after normalization to *TBP*. (**C**) Gene expression was expressed in relation to CTC count < vs. ≥ 5 CTC/7.5 mL. (**D**) *AR-V7* expression in cfRNA from plasma of patients with mHSPC (n = 4) and mCRPC (n = 38). Mean values ± SEM are indicated.

We next investigated the expression of our pre-selected gene panel in the plasma of our patient collective. To this end, cfRNA was isolated from plasma and cDNA was again amplified prior to qPCR. In contrast to the low expression in CTCs, the housekeeping genes *TBP* and *HPRT1* were detected at valuable Ct values in cfRNA plasma samples (Figure 5A) and were therefore normalized to TBP (Figure 5B). As expected, *CD45* expression was high in cfRNA from plasma with almost all samples (97%) being positive with a mean Ct value of 20.7. Overall, the expression of almost all genes, except that of *AR-FL* and *AR-V7*, was higher in plasma compared to CTCs (Figure 3B). Similar to the expression in CTCs, the expression of the analyzed genes was very heterogeneous with high expression levels of *AKR1C3*, *ERG*, and *TP53* (Figure 3C) but low expression of *AR-FL*, *AR-V7*, *KLK-2*, and *PSA*. *TMPRSS2* was detected in some of the plasma samples. We did not find any significant correlation of gene expression in plasma and CTC count. *AR-V7* was more highly expressed in mCRPC patients compared to mHSPC; however, the difference was not statistically significant (Figure 5D).

**Figure 6:**
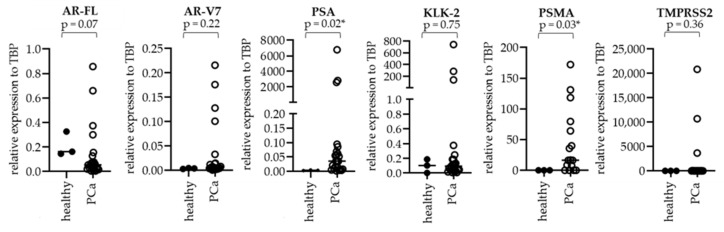
Gene expression in plasma-derived cfRNA from PCa patients compared to healthy donors. RNA was isolated from 23 plasma samples of patients with mPCa and 3 healthy donors. cDNA was amplified prior to qPCR. Gene expression is indicated in relation to *TBP*. Statistical comparisons are expressed with an asterisk (* *p* < 0.05).

Using cfRNA, we further addressed the question if any of the pre-selected genes were differently expressed in PCa patients compared to healthy donors. As shown in Figure 6, all healthy donor plasma samples (n = 3) were negative for *AR-V7*, *PSA*, *KLK-2*, *PSMA*, and *TMPRSS2*. All other genes showed a trend towards increased expression in mPCa patients compared to healthy controls. *PSA* and *PSMA* were significantly more highly expressed in mPCa patients compared to healthy controls (* *p* < 0.05).

**Figure 7:**
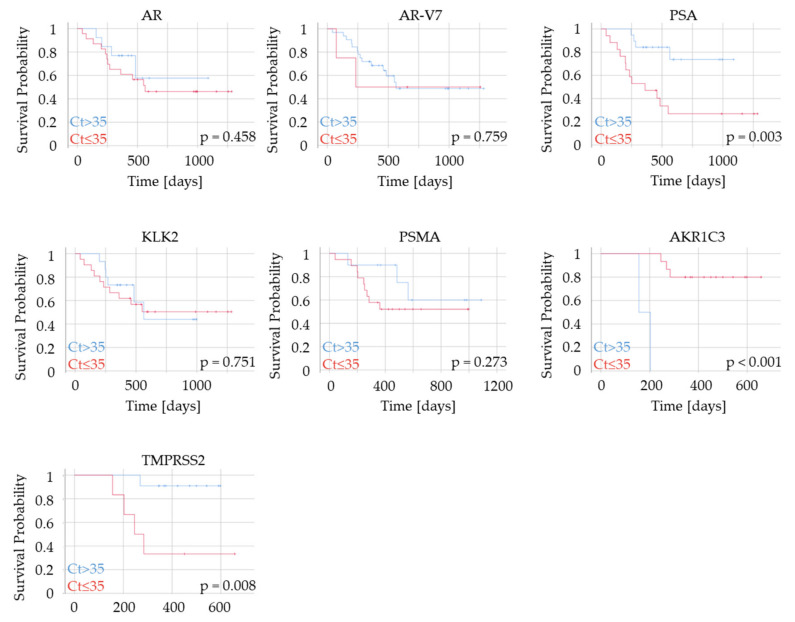
Association of plasma-derived cfRNA with survival probability in PCa patients. cfRNA was isolated from plasma of patients (n = 23) with mPCa, and Kaplan–Meier analysis was performed for expression status of *AR*, *AR-V7*, *PSA*, *KLK2*, *PSMA*, *AKR1C3*, and *TMPRSS2*.

We then performed Kaplan–Meier analyses to assess possible correlations between gene expression in PCa patients and overall survival (OS). High *PSA* as well as high *TMPRSS2* expression in plasma-derived cfRNA were associated with significantly shorter OS (* *p* < 0.05) (Figure 7). *AR*, *AR-V7*, *PSMA*, and *KLK2* were not significantly correlated with OS. Unexpectedly, high levels of *AKR1C3* were associated with a longer OS, although AKR1C3 protein expression has previously been linked with tumor progression [34].

### 3.4. Follow-Up of a Patient with mCRPC

**Figure 8:**
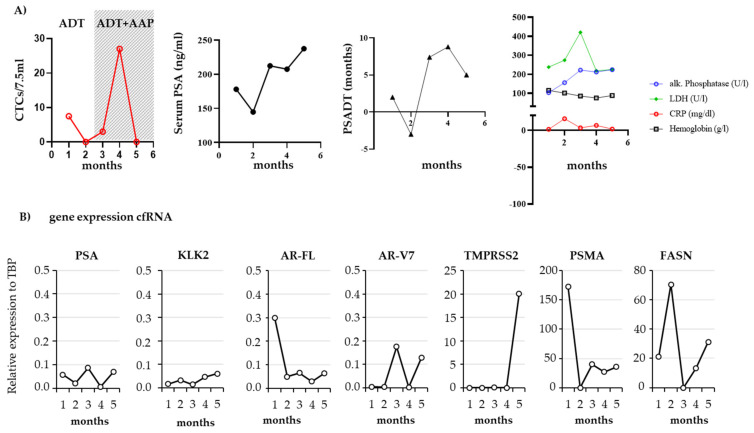
Monitoring CTC count and gene expression in a patient over 6 months. Five consecutive blood draws were taken from a 69-year-old patient with mCRPC who received first line androgen deprivation therapy (ADT) and radium-223. After tumor progression with disseminated bone metastases, second line therapy was started with abiraterone acetate and prednisone (AAP). (**A**) CTCs were enriched with Parsortix^TM^ and counted when positive for PSMA and/or panCK and negative for CD45. In addition, the following parameters were measured: serum PSA, PSADT, alkaline phosphatase, lactate dehydrogenase (LDH), C-reactive protein (CRP), and hemoglobin. (**B**) RNA was isolated from plasma and expression of genes was determined by qPCR. Gene expression was indicated in relation to *TBP*.

One of the patients was followed over a period of 6 months with five consecutive measurements of CTCs and plasma-derived cfRNA. This 69-year-old mCRPC patient received ADT and docetaxel as first line treatment after diagnosis of a primary mHSPC. After progression to mCRPC with disseminated bone metastases, the patient received radium-223 and ADT. Six months later, the first blood draw was taken while the patient still responded to the prior therapy. Another 2 months later, he was further treated with abiraterone acetate and prednisone (AAP) due to progressive disease. As shown in Figure 8A, CTC count and serum PSA dropped synchronously under ADT after radium-223 and increased again as an indicator of tumor progression, though AAP was started. In the beginning, CTC count increased to very high levels followed by a drop down to an undetectable level, indicating that CTC count correlated with treatment response. Nevertheless, CTC count did not reveal clinical progression as reflected by increasing PSA and alkaline phosphatase serum levels. Remarkably, *AR-V7* and *TMPRSS2* became detectable in the plasma of this patient during clinical progression. In addition, we observed that *PSMA* expression decreased following ADT treatment and increased again during clinical progression (Figure 8B). 

## 4. Discussion

In this study, we investigated the usefulness of plasma-derived cfRNA and CTCs for molecular analysis of a pre-defined gene panel. We used a very heterogeneous collective of mPCa patients, comprising different stages of progressive disease with various prior therapies as they generally appear in clinical routine examinations. The overall dropout rate in our study was 10%. Six out of the sixty-two blood samples could not be analyzed mostly due to heavy blood clotting, which is frequently observed in elderly patients with progressive disease. In 50% of our patients, we detected ≥5 CTCs/7.5 mL blood when using two marker-independent devices for CTC enrichment (ScreenCell and Parsortix^TM^). This number of CTCs has previously been defined as the critical threshold of CTC count that was associated with shorter overall survival in mCRPC patients [33]. In their study, de Bono et al. used the CellSearch system, which is based on the expression of EpCAM. However, when using different CTC enrichment devices, other CTC thresholds might also be more meaningful. Danila et al., for example, showed that >2 CTCs/7.5 mL blood was associated with worse survival [35]. Goodman et al. showed that 3 CTCs/7.5 mL was predictive for progression to CRPC in hormone-sensitive PCa patients [13]. Another study used a threshold of >4 CTCs/7.5 mL to demonstrate a shorter OS in mHSPC [36]. In our patient collective, 78.6% of patients had ≥1 CTCs/7.5 mL blood. Nevertheless, CTC count did not correlate with Gleason score or serum PSA, irrespective of using a threshold of 1 or ≥5 CTCs/7.5 mL. We have calculated various models (data not shown) using different CTC categories. However, none of those (e.g., CTCs as a continuous variable (HR 1.002, 95%CI 0.99–1.01) or as a categorical variable (CTS ≤5 or <5; HR 1.15, 95%CI 0.56–2.36)) found significant predictive value for death. Although there was a trend towards higher CTC counts in patients with mCRPC compared to mHSPC, the difference between the groups was not statistically significant. Similarly, higher CTC counts were measured in patients with tumor progression compared to those without. One possible reason for the failure to reveal any significant differences could be that our study cohort consisted of patients that were not previously selected based on defined characteristics. Consequently, we worked with a heterogeneous cohort of patients, with which clinicians are confronted during clinical routine in the outpatient department. Moreover, it must be considered that we used two different CTC enrichment devices, which may yield divergent CTC counts. In fact, mean CTC count in blood that was processed with the ScreenCell system was much higher (21 CTCs/7.5 mL) compared to Parsortix^TM^ (7 CTCs/7.5 mL). Although we cannot exclude that the number of CTCs in the Parsortix^TM^ group was generally lower, this suggests that when using CTC count as a clinically useful parameter, critical thresholds need to be adopted for each specific enrichment device and also for the markers used for the identification of CTCs.

Selecting the right marker that identifies a broad and representative spectrum of CTCs is challenging, in particular due to the heterogeneity of PCa. As mentioned before, the FDA-approved CellSearch system uses EpCAM and cytokeratin to identify CTCs. Other markers which are used to isolate CTCs from PCa patients are PSMA, PSA, PSCA (prostate stem cell antigen), and human glandular kallikrein 2 (hK2) (reviewed by [37]). In this study, we used PSMA and cytokeratin to identify CTCs. PSMA is a cell surface receptor, which is increased in all stages and grades of PCa and routinely used as a diagnostic and therapeutic target in the clinic [38,39,40,41,42], which is, however, also heterogeneously expressed among PCa cells [43]. We therefore simultaneously stained for intracellular panCK to increase the capture efficiency for CTC counting by immunofluorescence. This, in fact, resulted in increased capture rate in spike-in experiments with immortalized cell lines. Despite this, it should be considered that even cytokeratin may be lost in cells undergoing epithelial-mesenchymal transition (EMT) [44]. El-Heliebi et al., for instance, observed that not all CTCs were positive for panCK after processing the blood with Parsortix^TM^ [44], indicating that a substantial number of CTCs exhibit an active state of EMT. Hence, despite the combination of PSMA and panCK, we may underestimate the CTC count.

Using intracellular panCK for CTC identification further implies a decrease in RNA quality, which may significantly impair further molecular analysis. We, in fact, observed that the expression of most of our pre-selected genes was very low. In particular, *AR-FL*, *AR-V7*, *PSA*, and *KLK3* expression was very low. A possible reason for this could be our selection for PSMA-positive CTCs. A recent study showed that CRPC patients harbor CTCs with different degrees of *AR* activity [45]. Using quantitative immunofluorescence, these authors demonstrated that there is a population of CTCs with an “AR-off” status where *PSMA* is expressed but AR signaling is shut down as shown by lack of PSA expression. Another explanation may be the initial low amount of RNA for qPCR, since even the housekeeping genes could not be detected in the CTC samples. Therefore, we decided to indicate absolute Ct values. Of note, we used very low numbers of CTCs to isolate RNA for qPCR, which were specifically picked after staining for PSMA, panCK, and CD45 to get rid of significant contamination with CD45-positive cells after Parsortix^TM^ enrichment. The picking of single CTCs certainly brings the advantage that the result is not confounded by the pool of contaminating WBCs in the sample; however, is also rather challenging and requires manual experience as well as specific additional technologies. We were able to pick CTCs in 52% of the samples and showed that this strategy efficiently eliminated the contamination with white blood cells. However, with regard to improving RNA quality for molecular analysis, the use of intracellular panCK should be avoided. 

With regard to the technical challenges in the use of CTCs for molecular analysis, we investigated whether cfRNA from plasma could be used to determine gene expression. We decided to use cfRNA over cfDNA, since the analysis of cfRNA allows for measuring the amount of gene expression on the transcription level. A comparison of gene expression with healthy donors revealed that *PSA* and *PSMA* were significantly more highly expressed in mPCa patients. All other genes showed an insignificant trend towards increased expression in mPCa. Kaplan–Meier analysis further revealed that high *PSA* expression was significantly associated with poor overall survival. Moreover, low expression of *TMPRSS2* and *AKR1C3* was significantly associated with poor overall survival. This finding was unexpected and needs further investigation in a higher number of samples since previous findings showed that high AKR1C3 expression supports tumor progression [34,46,47,48] and antiandrogen resistance [49,50,51]. *AR*, *AR-V7*, *KLK-2*, and *PSMA* expression did not significantly correlate with survival in our patient cohort. *AR-V7* was detected in a small number of patients. A total of 16% of CTCs and 14% of plasma samples were positive for *AR-V7*. Importantly, AR-V7 was only detected in mCRPC but not in mHSPC, though the number of mHSPC patients was low. This low number of *AR-V7*-positive samples in our study cohort is in contrast to the results published by El-Heliebi et al. who found AR-V7 in 71% of CRPC patients [44]. These authors, however, used in situ hybridization instead of qPCR and detected AR-V7 in CTCs which were negative for cytokeratin. 

With regard to using CTCs or cfRNA to help with therapy decisions in mPCa, we monitored one patient with five consecutive blood draws over 6 months. In particular, the best follow-up regime after radium-223 treatment is still under discussion. Blood draw (PSA, ALP, LDH) and imaging (PET/CT) alone can be misleading, e.g., because of the so-called flare phenomenon [52]. Low levels of <5 CTCs/7.5 mL or a decline of >50% of CTCs from baseline has recently been reported as a biomarker of a good response after the third cycle of radium-223 treatment [53]. To date, the field of CTC counts in the follow-up after radium-223 is still under investigation, although we performed consecutive CTC and cfRNA measurements to investigate their role as possible biomarkers for long term treatment response. In this single patient series, CTC count failed to display the tumor progress, which was proven by PSA rise and radiographic and clinical progress. Notably, as mentioned before, expression of *AR-V7*, *TMPRSS2*, and *PSMA* increased in CTCs during disease progression.

## 5. Conclusions

Our study focused on the expression of a limited gene panel by qPCR in an unselected, very heterogeneous cohort of patient samples, similar to that with which clinicians are confronted in their routine work. This may explain why CTC count in our cohort did not correlate with any clinical parameter or the expression of any of the genes analyzed. In fact, gene expression analysis in plasma-derived cfRNA, which is technically less challenging than the use of CTCs, may be sufficient for clinical daily routine. Although our data would be strengthened by a higher patient number, they also highlight the difficulty of establishing a marker to monitor treatment efficacies in mCRPC patients. Further studies are under way to clearly define the molecular expression pattern of CTCs through RNA sequencing in order to obtain a more convincing gene panel in a defined cohort of patients with mPCa, which may be of clinical prognostic value.

## Figures and Tables

**Table 1:** Patients’ demographic clinical characteristics.

Total Number of Patient Samples	62
Median age, years (range)	71 (56–88)
ISUP grade (number of samples)	
1–3	13
4	13
5	36
Tumor stage (number of samples)	
mHSPC	8
High volume	7
High risk	7
mCRPC	54
Primary M1 PCa	36
Metastatic sites (number of samples)	
Lymph nodes	53
Bone	58
Liver	5
Lung	7
Other visceral mets	3
Blood analytes	
Median PSA, ng/mL (range)	46 (0.6–7014)
Median alkaline phosphatase, U/L (range)	95 (38–783)
Median LDH, U/L (range)	226 (133–1173)
Median CRP, mg/dL (range)	0.6 (0.1–13.3)
Median hemoglobin, g/dL (range)	12.3 (7.15.8)
Local treatment (number of samples)	
Radical prostatectomy	19
EBRT (external beam radiation)	5
LDR (low dose rate) brachytherapy	2
Therapies prior to sample collection (number of samples)	
ADT	61
Enzalutamide	22
Abiraterone	26
Docetaxel	41
Cabazitaxel	12
Radium-223	17
Lutetium^177^-PSMA	6

**Table 2:** Primer sequences.

PrimePCR PreAmp and PrimePCR Probe Assay (Biorad)		
Gene symbol	Gene name	Unique Assay ID
*AKR1C3*	Aldo-keto reductase family 1 member C3	qHsaCEP0040990
*AR-FL*	Androgen receptor (full-length)	qHsaCIP0026366
*CD45*	Tyrosine phosphatase receptor type C	qHsaCEP0041630
*ERG*	ETS-related gene	qHsaCEP0041582
*FASN*	Fatty acid synthase	qHsaCIP0026813
*KRT18*	Keratin 18	qHsaCEP0035862
*PSMA*	Prostate-specific membrane antigen	qHsaCEP0049804
*TMPRSS2*	Transmembrane Serine Protease 2	qHsaCIP0028919
*TP53*	Tumor protein P53	qHsaCEP0052284
**PreAmp Primer**		
Gene	Primer forward (F), Primer reverse (R)	
*AR-FL*	F: ACATCAAGGAACTCGATCGTATCA, R: GGGCACTTGCACAGAGATGA	
*AR-V7*	F: AAGAGCCGCTGAAGGGAAAC, R: TCCAGACTATCCACTAGAGCCC	
*HPRT1*	F: ACACTGGCAAAACAATGCAGA, R: AGTCAAGGGCATATCCTACAACAA	
*KLK-2*	F: TCAGAGCCTGCCAAGATCAC, R: TTTACCACCTGTCCAGAGCC	
*PSA*	F: AGTGCGAGAAGCATTCCCAA, R: AAGCTGTGGCTGACCTGAAA	
*TBP*	F: GCCGAATATAATCCCAAGCGG, R: TTAGCTGGAAAACCCAACTTCTG	
**PCR Primer**		
*AR-FL*	F: CTGCTCAAGACGCTTCTA, R: ATCATTTCCGGAAAGTCCA, P (Probe): TCCGTGCAGCCTATTGCGAG	
*AR-V7*	F: GTCCATCTTGTCGTCTTC, R: GCAAGTCAGCCTTTCTTCA, P: GGGAGAAAAATTCCGGGTTGGC	
*HPRT1*	F: GCTTTCCTTGGTCAGGCAGTA, R: GTCTGGCTTATATCCAACACTTCGT, P: TCAAGGTCGCAAGCTTGCTGGTGAAAAGAA	
*KLK-2*	F: GACCACCTGCTACGCCTCAG, R: GGACAGGAGATGGAGGCTCA, P: ACCAGAGGAGTTCTTGCGCCCCA	
*PSA*	F: GTCTGCGGCGGTGTTCTG, R: TGCCGACCCACGAAGATC, P: CACAGCTGCCCACTGCATCAGGA	
*TBP*	F: CACGAACCACGGCACTGATT, R: TTTTCTTGCTGCCAGTCTGGAC, P: TCTTCACTCTTGGCTCCTGTGCACA	

**Table 3:** CTC count after enrichment with ScreenCell and Parsortix^TM^.

	Total	ScreenCell	Parsortix^TM^
Samples analyzed, n	56	31	25
Mean CTC count/7.5 mL (SEM)	15.1 (±4.5)	21.2 (±7.6)	7.4 (±1.8)
CTC count ≥ 1/7.5 mL, n (%)	44 (78.6%)	24 (77.4%)	20 (80.0%)
CTC count ≥ 5/7.5 mL, n (%)	28 (50.0%)	17 (54.8%)	11 (44.0%)

**Table 4:** Number of CTCs picked upon PSMA and/or panCK positivity and CD45 negativity through immunofluorescence.

Patient	Method	PSA (ng/mL)	CTCs/7.5 mL	Number of CTCs Picked
47	Parsortix+ALS	144.8	0	0
51	Parsortix+ALS	2.7	0	0
56	Parsortix+ALS	26.8	1.5	0
58	Parsortix+ALS	10.9	1.5	0
59	Parsortix+ALS	33.8	0	0
61	Parsortix+ALS	9.0	0	0
62	Parsortix+ALS	237.5	0	0
64	Parsortix+ALS	32.9	1.5	0
66	Parsortix+ALS	67.91	7.5	0
67	Parsortix+ALS	232.6	7.5	0
55	Parsortix+ALS	212.3	3.0	1
63	Parsortix+ALS	23.2	3.0	2
50	Parsortix+ALS	22.3	7.5	3
57	Parsortix+ALS	162.3	18.0	4
46	Parsortix+ALS	178.1	7.5	5
65	Parsortix+ALS	182.1	12.0	8
49	Parsortix+ALS	6.0	21.0	12
60	Parsortix+ALS	207.4	27.0	18
43b	Parsortix+ALS	113.6	33.0	22

## Data Availability

The data presented in this study are available on request from the corresponding author.

## Supplementary Materials

The following are available online at https://www.mdpi.com/article/10.3390/biomedicines9081004/s1. Figure S1: CTC count in mPCa patients with different prior therapies, including androgen deprivation therapy (ADT), docetaxel (doce), enzalutamide (enza), abiraterone acetate (abi), radium-223 (rad), and Lutetium177-PSMA- therapy (LuPSMA). Blood was enriched with ScreenCell or ParsortixTM and counted when positive for PSMA and/or panCK and negative for CD45. Mean values with SEM are shown.
