# Supplementary material for: Validation of Cell-Free RNA and Circulating Tumor Cells for Molecular Marker Analysis in Metastatic Prostate Cancer
Source: Biomedicines. 2021 Aug 12;9(8):1004. doi: 10.3390/biomedicines9081004 (PMC8391593; doi:10.3390/biomedicines9081004)
Supplement: Supplementary file 1 [file biomedicines-09-01004-s001.zip › biomedicines-1339432-supplementary.pdf]

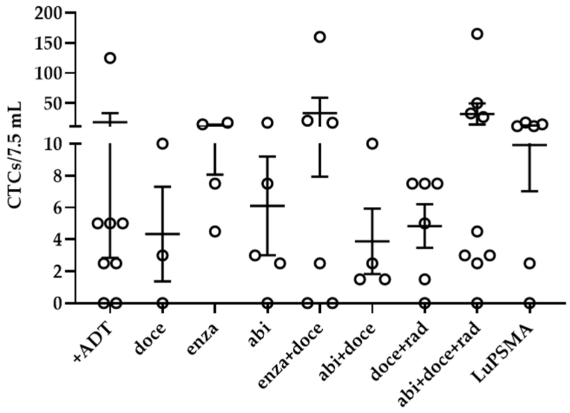

Supplementary Figure 1. CTC count in mPCa patients with different prior therapies, including androgen deprivation therapy (ADT), docetaxel (doce), enzalutamide (enza), abiraterone acetate (abi), radium-223 (rad), and Lutetium^{177-}PSMA- therapy (LuPSMA). Blood was enriched with ScreenCell or Parsortix™ and counted when positive for PSMA and/or panCK and negative for CD45. Mean values with SEM are shown.
